# Convolution kernel and iterative reconstruction affect the diagnostic performance of radiomics and deep learning in lung adenocarcinoma pathological subtypes

**DOI:** 10.1111/1759-7714.13161

**Published:** 2019-08-19

**Authors:** Wei Zhao, Wei Zhang, Yingli Sun, Yuxiang Ye, Jiancheng Yang, Wufei Chen, Pan Gao, Jianying Li, Cheng Li, Liang Jin, Peijun Wang, Yanqing Hua, Ming Li

**Affiliations:** ^1^ Department of Radiology Huadong Hospital Affiliated to Fudan University Shanghai China; ^2^ Diagnosis and Treatment Center of Small Lung Nodules of Huadong Hospital Shanghai China; ^3^ Diannei Technology Shanghai China; ^4^ Department of Electronic Engineering Shanghai Jiao Tong University Shanghai China; ^5^ SJTU‐UCLA Joint Center for Machine Perception and Inference Shanghai Jiao Tong University Shanghai China; ^6^ GE Healthcare Beijing China; ^7^ Department of Radiology Tongji Hospital, School of Medicine, Tongji University Shanghai China; ^8^ Institute of Functional and Molecular Medical Imaging Fudan University Shanghai China

**Keywords:** Convolution kernel, deep learning, iterative reconstruction, lung adenocarcinomas, radiomics

## Abstract

**Background:**

The aim of this study was to investigate the influence of convolution kernel and iterative reconstruction on the diagnostic performance of radiomics and deep learning (DL) in lung adenocarcinomas.

**Methods:**

A total of 183 patients with 215 lung adenocarcinomas were included in this study. All CT imaging data was reconstructed with three reconstruction algorithms (ASiR at 0%, 30%, 60% strength), each with two convolution kernels (bone and standard). A total of 171 nodules were selected as the training‐validation set, whereas 44 nodules were selected as the testing set. Logistic regression and a DL framework‐DenseNets were selected to tackle the task. Three logical experiments were implemented to fully explore the influence of the studied parameters on the diagnostic performance. The receiver operating characteristic curve (ROC) was used to evaluate the performance of constructed models.

**Results:**

In Experiments A and B, no statistically significant results were found in the radiomic method, whereas two and six pairs were statistically significant (*P* < 0.05) in the DL method. In Experiment_C, significant differences in one and four models were found in the radiomics and DL methods, respectively. Moreover, models constructed with standard convolution kernel data outperformed that constructed with bone convolution kernel data in all studied ASiR levels in the DL method. In the DL method, B0 and S60 performed best in bone and standard convolution kernel, respectively.

**Conclusion:**

The results demonstrated that DL was more susceptible to CT parameter variability than radiomics. Standard convolution kernel images seem to be more appropriate for imaging analysis. Further investigation with a larger sample size is needed.

## Introduction

Lung cancer is one of the leading causes of cancer‐related death both in males and females.^1^ How to achieve an early and better prognosis has constantly received enormous attention of researchers in the past decades. In the battle of fighting lung cancer, medical imaging modalities including computed tomography (CT), magnetic resonance (MR), and positron emission tomography (PET) have played a critical role and infiltrate each process of clinical practice such as disease detection, evaluation, diagnosis, surveillance.^2^ Buoyed by the recent innovations in statistical methodology and imaging data‐driven technologies, more and more researchers agree that diversified medical images encompass not only hand‐crafted descriptors which could be easily evaluated by radiologists' eyes, but also innumerable agnostic representatives reflecting the tumor heterogeneity, biological process or gene‐expression patterns.^3^, ^4^, ^5^ On the basis of this theory, utilizing these invisible features derived from routine medical images to diagnose disease, monitor treatment response and make tailored therapy, has sprung up rapidly with the initial intention to facilitate precision medicine.

Radiomics, referring to converting imaging data into mineable high‐dimensional data with the use of large number of automatic algorithms, is one of the emerging imaging analysis strategies.^6^ Radiomics‐based researches have been increasingly applied to various clinical contexts and outperformed previous studies in several domains, including lung cancer domain.^7^, ^8^ Of note, while clinically promising, radiomics‐based analysis has initially struggled with many challenges related to technical variability across imaging modalities, scanners, and parameters, highlighting that the published results or models in clinical practice due to the replicability should be carefully utilized.^9^, ^10^ Several studies have investigated the influence of different CT acquisition parameters on radiomic features, e.g., thickness, radiation dose, and reconstruction algorithm.^9^, ^10^, ^11^, ^12^ However, only a few studies have investigated the test‐retest analyses on patients due to the ethical and logistical issues, whereas phantoms and different materials were usually used as the surrogate subjects.^9^, ^10^ Therefore, substantial discrepancies between real lung nodules and phantoms may limit the clinical utility of radiomics. Moreover, how the variability among parameters affects the diagnostic performance is yet to be investigated in depth.

Deep learning, a subset of machine learning that contains sophisticated sets of algorithms, has recently made substantial strides in interpreting and approximating very complex data and is starting to take off in medical and radiology fields.^2^, ^13^, ^14^ It has been reported that deep learning has already matched and even outperformed humans in task‐specific applications,^15^, ^16^ including medical applications.^17^, ^18^ These encouraging research results are because of recent advances in artificial intelligence (AI) research, and the massive amounts of data now available to train algorithms and modern, powerful computational hardware. It is important to note that the quality of data, including the sample size, homogeneity, integrity and purity, is the lifeline of deep learning. Data whitening and normalization, essential data preprocessing steps, may mitigate the influence of the non‐normalization data in deep learning.^3^ However, little is known about whether and to what extent the CT scanning parameters affect the performance and robustness of deep learning. To the best of our knowledge, no previous studies have investigated the issue.

The standardization of CT parameters in image processing research has increasingly received attention. The same slice thickness, manufacture, and scanning parameters are carefully applied in current clinical researches, with the aim being to obtain more reliable and reproducible results. However, some parameters still need to be tailored for patients due to their specific clinical purpose (e.g., dose reduction) and necessity of the study itself. In view of this, we aimed to carry out a clinical study to investigate the influence of convolution kernel and adaptive statistic iterative reconstruction (ASiR GE Healthcare, Waukesha, Wisconsin) on the diagnostic performance of radiomics and deep learning in lung adenocarcinomas.

## Methods

This study was approved by the Institutional Review Board (Grant No.2017K062), which waived the requirement for patients' informed consent referring to the CIOMS guideline.

### Patients

A total of 284 consecutive patients with suspected lung cancers from November 2017 to July 2018 were selected as eligible to undergo the same scanning parameters on one CT scanner in our institution. The inclusion criteria were: (1) Patients who had undergone thin‐slice chest CT imaging, (2) patients with pathologically confirmed lung adenocarcinoma and its precancer status and (3) patients with no treatment prior to surgery. Exclusion criteria were: (1) Pulmonary nodules without pathological diagnosis, (2) pulmonary nodules pathologically confirmed with other kinds of lung cancers and (3) patients who had received treatment prior to surgery. Finally, a total of 183 patients (118 female and 65 male; mean age, 56.4 years ± 13.0 [standard deviation]; range, 23–92 years) with 215 pathologically confirmed pulmonary nodules (26 patients had more than one nodule) were enrolled in the study. The average size of included nodules was 13.74 mm ± 9.81 (standard deviation), range, 3.61–70.77 mm. According to 2011 new classification, nodules were divided into four categories: atypical adenomatous hyperplasia (AAH), adenocarcinomas in situ (AIS), minimally invasive adenocarcinomas (MIA) and invasive adenocarcinomas (IAC). We randomly divided the nodules into two independent sets: 171 lesions constituted the training and validation set, whereas 44 lesions constituted the testing set. The distribution of our study population is summarized in Table 1.

**Table 1:** Number of nodules for training, validation, and testing

Groups	Training and validation	Testing	Total
AAH	0	1	1
AIS	19	5	24
MIA	86	21	107
IAC	66	17	83
AAH‐AIS‐MIA	105	27	132
Total	171	44	215

### CT imaging acquisition

All studies were performed with a 64‐detector row CT scanner (Discovery CT750 HD; GE Healthcare, Waukesha, WI, USA) with the following acquisition parameters: 40 mm (64 × 0.625 mm) detector collimation, 120 kVp tube voltage, automatic tube current modulation (Smart mA, GE Healthcare) to achieve a preset noise index of 12 HU (corresponding thickness: 5 mm), 0.5 second rotation time; 50 cm scan field of view and 512 × 512 imaging matrix. All images were reconstructed with a section thickness of 1.25 mm (corresponding NI: 24HU) by using three reconstruction algorithms: ASiR at 0% strength, ASiR at 30% strength, and ASiR at 60% strength each with two convolution kernels: bone and standard. ASiR, iteratively refining each pixel value measured with filtered back projection (FBP) to an idealized estimate, is an image reconstruction algorithm introduced for CT in 2008, with gains in image quality compared to noise filtering techniques.^19^, ^20^ Consequently, all the CT imaging data was divided into six groups (ASiR at 0% strength and bone, B0; ASiR at 30% strength and bone, B30; ASiR at 60% and bone, B0; ASiR at 0% strength and standard, S0; ASiR at 30% strength and standard, S30; ASiR at 60% and standard, S60). Images with 1.25 mm (NI = 24 HU) thickness were used for analysis.

### Data annotation and preparation

The ASiR 30% with standard kernel images (good for presenting the tumor‐lung boundary) were selected as reference image set for delineation based on our experience. One radiologist with five‐years experience in chest CT imaging manually and independently delineated all included nodules at the voxel level using a medical image processing and navigation software 3D Slicer (version 4.8.0, Brigham and Women's Hospital). The volume of interests (VOIs) of nodules were subsequently confirmed (modified or redelineated) by another radiologist with 12‐years experience in chest CT imaging. The image mask was propagated to all other image sets such that all images had the same annotation applied. Each segmented nodule was assigned a specific pathological label (AAH, AIS, MIA and IAC) based on pathological report. Due to the unbalance distribution of four categories (AAH = 1, AIS = 24, MIA = 107, IAC = 83), dividing the data set into preinvasive lesions (AAH + AIS = 25) and invasive lesions (MIA + IAC = 190) was clearly too low for fairly training the radiomic models or the deep neural networks. To avoid overfitting, the study merged the samples labelled as AAH, AIS and MIA into a single class “AAH‐AIS‐MIA”, also named as non‐IAC class. Fortunately, it is still reasonable in the clinical context, and these three subtypes of lesions (≤3 cm) are reported to have a 100% or near 100% disease‐specific survival, if completely resected.^21^ Therefore, a binary classification of IAC nodules and non‐IAC nodules was used in the further training and validation.

### Invasiveness prediction with radiomics in different parameter groups

Pyradiomics,^22^ an open source python toolkit for extracting radiomic features, was used to extract a total of 1301 features from first order statistics, shape‐based features, gray level co‐occurrence matrix (GLCM), gray level run length matrix (GLRLM), gray level size zone matrix (GLSZM), neighboring gray tone difference matrix (NGTDM), gray level dependence matrix (GLDM) in our study. Detailed information on these predefined features is described in Supplementary Information.

The principal component analysis (PCA) was used to perform the dimension reduction and avoid the overfitting problem. Logistic regression with L2 regularization (to avoid the overfitting problem) was used to model the relation between the radiomic features and invasiveness of lung adenocarcinomas with the widely used scikit‐learn library in Python.^23^ Ten‐fold cross validation search was performed on the training set with 1 000 randomly sampled values of regularization term C. To fully explore the influence of convolution kernel and ASiR on diagnostic performance of radiomics in lung adenocarcinomas, three logical experiments were investigated in our study:

Experiment_A (Exp_A): constructing and testing the model in six groups individually (controlling the parameter as the same for both model and tested data);

Experiment_B (Exp_B): constructing the model by using all six groups training data (*n* = 215*6 = 1290), and testing the model on six groups tested data individually (controlling the model as the same);

Experiment_C (Exp_C): constructing the model by using six groups training data individually, then testing one of the six models on the rest of five groups tested data and testing five different models on the rest of one group tested data (cross‐testing).

Three experiments are illustrated in the Supporting information. Invasiveness Prediction with Deep Learning in Different Parameter Groups.

In this study, a deep learning framework based on DenseNets^24^ with a multi‐task strategy, which was proposed in our previous study^18^ and could simultaneously and automatically predict IAC nodules and non‐IAC nodules, as well as nodule segmentation masks, was selected to model the task. The multi‐task design was inspired by considering the medical relevance of the two tasks‐classification (IAC, non‐IAC) and nodule segmentation. Of note, the segmentation worked as an auxiliary task in the multi‐task learning architecture, with the aim to guide the network to attend the nodule part. Such an ingenious architecture gave DenseNets the ability to perform the classification and segmentation tasks end‐to‐end efficiently.

Briefly, the input of the multi‐task model was a 3D cubic patch of 48 × 48 × 48 mm (voxels, 1 voxel denotes 1 mm), generated by a (preprocessed) chest CT scan and the position c = (z, y, x), i.e., the mass center (roughly) of the nodule, and the output was the categorical probability for the two categories (IAC, non‐IAC), as well as the model‐generated mask of the nodule segmentation. The preprocessing followed “standard” procedure for chest CT: the input CT scans were converted into Hounsfield units, followed by resizing of volumetric data into spacing of 1 × 1 × 1  mm by trilinear interpolation, clipping the voxel intensity into I_HU_ ∈ [−1024, 400], quantifying the density into grayscale, and transforming the values to I ∈ [−1, 1) by a mapping $I=\left\lfloor \frac{I_{\mathrm{HU}}+1024}{400+1024}\times 255\right\rfloor /128-1$. During the training process, several data augmentation techniques (random offset, rotation, flipping, slight affine transformation) were used to increase the training data size and regulate the model.^18^ The multi‐task model had two output heads. The classification head could enforce the network to extract salient representatives for diagnosis and the segmentation head was able to teach the networks to attend the VOIs. The multi‐task loss could be expressed as *l*_*multi*_,

$$l_{\text{multi}}={\mathrm{al}}_{\mathrm{cls}}+{\mathrm{bl}}_{\mathrm{seg}}$$

*lcls* was binary cross‐entropy loss of the classification task, and lseg was dice loss of the segmentation task. The study chose a = 0.7, b = 0.3, since classification worked as a main task and segmentation works as an auxiliary task. When training the networks, all the network parameters were well initialized using “he uniform” method.^25^ To avoid potential bias, the proposed neural network in the study did not pretrain on any database. During optimization, the study sampled the training data with a ratio of 1: 1 for the two classes with a batch size of eight.

The network was implemented by using Python 3.6 based on TensorFlow 1.4.0^26^ and Keras 2.1.5^27^ deep learning library and trained the neural networks on a workstation with 1 NVIDIA GeForce GTX 1080 Ti GPU. The detailed training process is presented in Supplementary information.

Again, the aforementioned three experiments (Exp_A, Exp_B and Exp_C) were investigated by using the proposed deep learning system.

### Statistical analysis

The receiver operating characteristic curve (ROC) was used to evaluate the performance of constructed models. The ROC comparison analysis was performed to assess the statistical differences between AUCs by using the method developed by DeLong *et al*.^28^ *P* < 0.05 was considered statistically significant. All statistical analysis was performed with MedCalc 18.2.1.

## Results

### Experiment_A: Results of radiomics and deep learning

**Figure 1:**
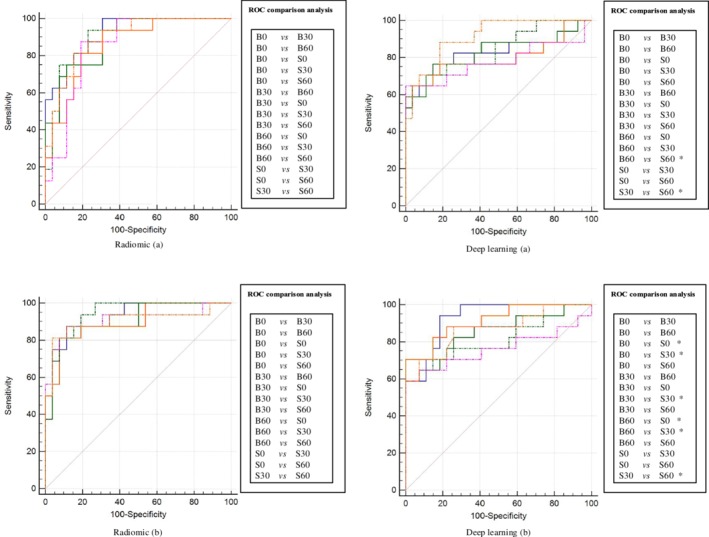
The two methods' results of ROC comparison analysis in Exp_A and Exp_B. The performance of six models constructed with two methods was performed with ROC comparison analysis in Exp_A and Exp_B. There were 15 pairs ROC comparison analysis in each method, specifically depicted in the right of the AUCs. The significant differences are marked as *. (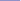) B0, (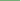) B30, (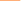) B60, (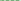) S0, (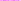) S30, (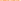) S60.

To investigate whether the studied parameters could affect the invasiveness prediction performance of radiomics and deep learning, we first individually constructed six models based on six groups using 171 nodules of training and validation set and tested the invasiveness prediction performance on corresponding group using 44 nodules of testing set, also named as Exp_A (controlling the parameter as the same for both model and tested data). While presenting differences between six models in the radiomic method, in term of the predicting performance (AUC value), no statistically significant results were found after performing the ROC comparison analysis. In contrast, the differences in predicting performance between B60 and S60, as well as between S30 and S60, were statistically significant in ROC comparison analysis when using the deep learning method (*P* < 0.05) (Table 2, Fig 1). Note that a trend demonstrated that models constructed by radiomic method outperformed those constructed by the deep learning method; model S60 was an exception. However, no statistical differences were found after comparing the AUCs in the two methods (Table 2).

**Table 2:** The invasiveness predicting performance of radiomics and deep learning in Exp_A and Exp_B

Model	AUC (radiomics)	AUC (deep learning)	*P*
Exp_A			
B0	0.928	0.830	0.1734
B30	0.863	0.828	0.632
B60	0.874	0.800	0.3914
S0	0.919	0.845	0.3006
S30	0.911	0.780	0.144
S60	0.885	0.911	0.6508
Exp_B			
B0	0.950	0.928	0.5972
B30	0.941	0.850	0.1906
B60	0.948	0.913	0.4553
S0	0.930	0.810	0.1311
S30	0.928	0.763	0.0653
S60	0.908	0.868	0.5848

*P* < 0.05 was considered statistically significant.

### Experiment_B: Results of radiomics and deep learning

The results of Exp_A appeared to draw the conclusion that the studied parameters could only affect the invasiveness prediction performance of the deep learning method. To further verify the assumption, we implemented the Exp_B, testing the predicting performance of the model fed by six groups mixed‐parameters data (controlling the model as the same) on six groups testing data. Integrating six groups data into one mixed‐parameters group and then modeling on it may be more appropriate in real clinical practice context, considering that substantial variability among CT scanning parameters were presented in different institutions. With regard to the radiomic method, the obtained results in Exp_B were surprisingly similar to that in Exp_A, which depicted that no statistically significant results were found after performing ROC comparison analysis. In terms of the deep learning method, as we expected, more pairs in ROC comparison analysis (six pairs vs. two pairs in Exp_A) were statistically significant (*P* < 0.05) (Table 2, Fig 1). Again, the performance of models constructed by the radiomic method were potentially superior to that of models constructed by the deep learning method (Table 2). However, the performance of two methods had no statistical differences.

### Experiment_C: Results of radiomics and deep learning

**Figure 2:**
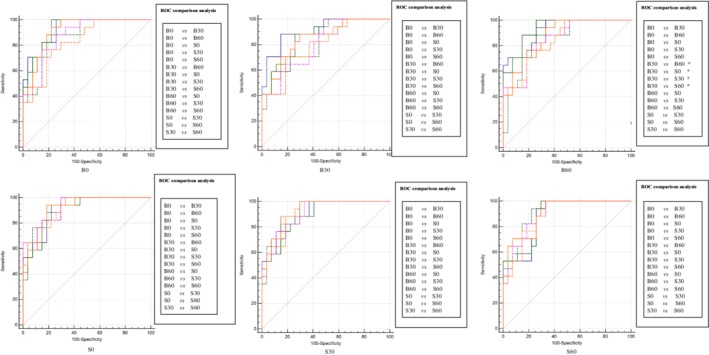
The radiomics method's results of ROC comparison analysis in Exp_C when comparing the AUCs of testing one model on six testing data sets. The performance of six models constructed with two methods was performed with ROC comparison analysis. There were 15 pairs ROC comparison analysis in each method, specifically depicted in the right of the AUCs. The significant differences are marked as *. Note that the phrases such as B0 below the AUCs represent the corresponding model. (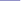) B0, (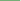) B30, (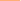) B60, (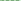) S0, (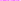) S30, (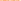) S60.

**Figure 3:**
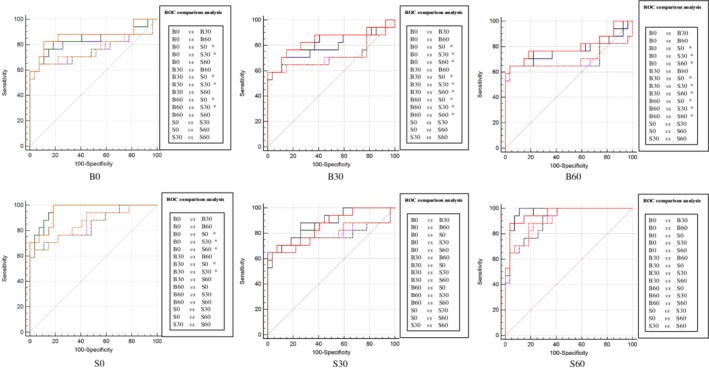
The deep learning method's results of ROC comparison analysis in Exp_C when comparing the AUCs of testing one model on six testing data sets. The performance of six models constructed with two methods was performed with ROC comparison analysis. There were 15 pairs ROC comparison analysis in each method, specifically depicted in the right of the AUCs. The significant differences are marked as *. Note that the phrases such as B0 below the AUCs represent the corresponding model. (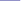) B0, (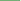) B30, (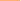) B60, (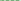) S0, (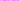) S30, (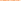) S60.

**Figure 4:**
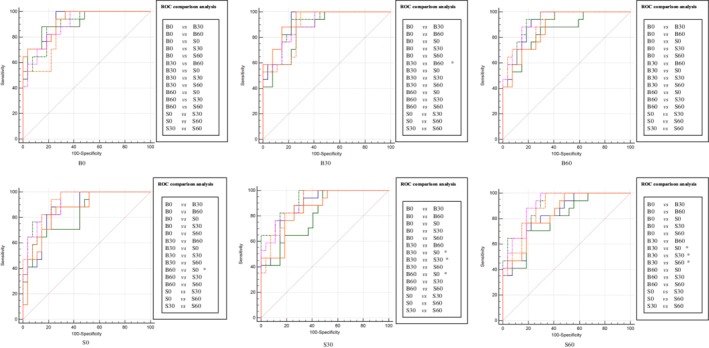
The radiomics method's results of ROC comparison analysis in Exp_C when comparing the AUCs of testing six models on one testing data set. The performance of six models constructed with two methods was performed with ROC comparison analysis. There were 15 pairs ROC comparison analysis in each method, specifically depicted in the right of the AUCs. The significant differences are marked as *. Note that the phrases such as B0 below the AUCs represent the corresponding testing data set. (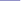) B0, (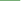) B30, (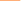) B60, (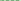) S0, (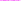) S30, (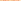) S60.

**Figure 5:**
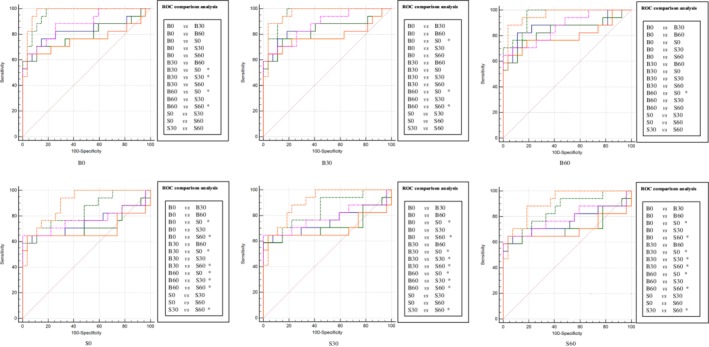
The deep learning method's results of ROC comparison analysis in Exp_C when comparing the AUCs of testing six models on one testing data set. The performance of six models constructed with two methods was performed with ROC comparison analysis. There were 15 pairs ROC comparison analysis in each method, specifically depicted in the right of the AUCs. The significant differences are marked as *. Note that the phrases such as B0 below the AUCs represent the corresponding testing data set. (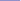) B0, (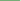) B30, (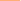) B60, (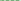) S0, (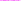) S30, (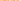) S60.

Inspired by the results of Exp_A and Exp_B, we hypothesized that more striking distinctions would be presented after performing Exp_C, constructing the model by using six groups training data individually, and testing one of the six models in the rest of five groups tested data or testing five different models in the rest of one group tested data (cross‐testing). Exp_C could actually be considered as a strategy to investigate the generalization ability of a model. Among the six models, one radiomics‐based model and four deep learning‐based models we found significant differences in ROC comparison analysis (Figs 2 and 3). Of note, there were eight pairs in four testing data sets (radiomics method) and 33 pairs in six testing data sets (deep learning method) were statistically significant after comparing the AUCs of different models (Figs 4 and 5). These findings further supported the results of Exp_A and Exp_B.

After comprehensively and thoroughly analysing the results, it was not hard to come to several conclusions. Firstly, the cross‐testing performance of bone‐kernel models (B0, B30 and B60) and S30 constructed by radiomic method outperformed the corresponding performance of models constructed by the deep learning method (Table 3). Moreover, the testing performance of model B60 on B0 data set had significant difference between two methods (Table 3 marked as *). Secondly, standard‐kernel models (S0, S30 and S60) constructed by the deep learning method were more stable and generalized, since the models constructed on S30 and S60 groups found no significant differences in ROC comparison analysis (Fig 3) and an incremental performance was presented after testing these models on bone‐kernel groups (Table 3). Thirdly, focusing on ASiR, B0 and S60 seemed to have the best performance in bone‐kernel and standard‐kernel models, respectively when performing the deep learning method (Table 3). This trend was analogous to the results depicted in Exp_A and Exp_B. Lastly, when we compared the performance of different convolution kernel models by controlling the ASiR level as the same, an interesting circumstance was observed. Models constructed with standard convolution kernel data outperformed those constructed with bone convolution kernel data in all studied ASiR levels – 0%, 30% and 60% in deep learning method. A similar trend was also found in the radiomic method, except for testing on the B0 and bone 30 data set (Table 3).

**Table 3:** The invasiveness prediction performance of radiomics and deep learning in Exp_C

AUC (model)	B0	B30	B60	S0	S30	S60
Radiomics						
AUC (B0)	0.928	0.930	0.915	0.869	0.876	0.830
AUC (B30)	0.911	0.863	0.847	0.828	0.810	0.800
AUC (B60)	0.924*	0.935	0.874	0.852	0.852	0.845
AUC (S0)	0.904	0.906	0.919	0.919	0.926	0.911
AUC (S30)	0.898	0.893	0.926	0.915	0.911	0.911
AUC (S60)	0.880	0.885	0.906	0.915	0.898	0.885
Deep learning						
AUC (B0)	0.830	0.845	0.869	0.749	0.752	0.760
AUC (B30)	0.793	0.828	0.839	0.715	0.715	0.712
AUC (B60)	0.765*	0.786	0.800	0.691	0.695	0.702
AUC (S0)	0.961	0.961	0.954	0.845	0.852	0.861
AUC (S30)	0.880	0.874	0.867	0.769	0.780	0.784
AUC (S60)	0.972	0.963	0.959	0.902	0.906	0.911

* Indicated statistically significant difference between two AUC values (0.924 vs 0.765). B0 in parentheses in the first column was the name of corresponding models and B0 in first row represents the corresponding testing data set. This explanation applies to all models and data sets.

## Discussion

Radiomics and deep learning are the most frequently used imaging analysis strategies in radiology discipline. However, a non‐negligible drawback faced by both strategies is that the diagnostic performance is susceptible to CT scanning parameters, and therefore it might limit their use in clinical practice. To tackle this issue, we have implemented three logical experiments to investigate the influence of two representative CT parameters: convolution kernel and strength of iterative reconstruction (ASiR in specific), on the diagnostic performance of radiomics and deep learning in lung adenocarcinomas. The current study has demonstrated that the invasiveness prediction performance of deep learning was more likely to be affected by the convolution kernel and the strength of ASiR and inferior to radiomics. Moreover, models constructed with standard convolution kernel data potentially outperformed those constructed with bone convolution kernel data, especially in the deep learning method.

It is well established and accepted that the variability due to CT scanning parameters is one of the vital factors affecting the robustness and reproducibility of radiomic features. On the contrary, deep learning, characterized by minimizing the efforts and optimizing the efficiency, is generally and initially expected to achieve a better and more stable performance than radiomics with respect to the task studied in the research. However, an opposite conclusion has been drawn in the current study. This may be partially explained by the insufficient data included in our study. Deep learning is a data‐driven strategy which requires a large amount of data in the whole process of training and validation, as well as testing. Insufficient data may cause the inadequate learning by the deep learning networks, followed by the inferior performance than radiomics and unstable diagnostic performance. On the other hand, ASiR, one of the studied parameters, may actually have relatively less impact on the robustness of radiomic features than other previous studied parameters, such as thickness, noise index and tube current, which has already been proved to substantially affect the robustness of radiomic features by affecting pixel‐by‐pixel intensity variability.^10^, ^29^ Research performed by Abhishek *et al*.^9^ concluded that radiomic features were influenced by acquisition parameters (noise index and tube current) more than ASiR. The study by Justin *et al*. also demonstrated that ASiR affected fewer radiomic features than radiation dose.^30^ Moreover, each nodule in the six different groups used the identical segmentation mask for further analysis in our study; substantially eliminating the variability related to the segmentation. This approach may also mitigate the effect on the radiomic features. In this context, the radiomic method may desrve the better performance and more stable results.

Note that no significant differences were found between models for radiomics method in Exp_A and Exp_B, whereas Exp_C demonstrated significant differences. This may be explained by the following: (1) In Exp_A, since the diagnostic performance was evaluated by testing one parameter model on the same parameter tested data, the constructed six models could learn the respective important representatives for differentiating non‐IAC from IAC, and thus obtain similar performance; even the value of extracted features is different in six groups data. Therefore, the differences between models are either absent or not so obvious. (2) In Exp_B, the mixed model was trained by the data from the six groups and may potentially learn all the important representatives for differentiating non‐IAC and IAC in each parameter data. In this context, when we tested the mixed model on six group tested data, it was also difficult to see the differences (e.g., in the radiomics method). However, the differences would be more obvious than Exp_A due to the mixed data for training the model, which was proven in the deep learning method. (3) In Exp_C, we tested models fed by one parameter on another tested parameter data, considered as a strategy to evaluate the generalization ability. In this way, the tested data with a specific parameter was really unknown to its tested model and the important representatives in one parameter data may not so vital for differentiating non‐IAC form IAC in another. Therefore, the differences were without doubt obvious compared with Exp_A and Exp_B, which was proved in both the radiomics and deep learning methods.

Instead of using high‐pass filter algorithms such as bone convolution kernel, standard convolution kernel, which uses low‐pass filter algorithms, reduces the higher frequency contribution with decreasing noise and spatial resolution.^31^ Imaging noise may play a vital role in influencing the extraction of radiomic features and confusing the representatives learned by deep learning networks, indicating that a smoother reconstruction algorithm (i.e., standard convolution kernel) is supposed to achieve better reputation in stability and reproductivity. Previous studies have shown that smoother reconstruction algorithms are more favorable for reproducibly extracting quantitative features^32^ and perform better in diagnosis.^33^ Our study obtained the same results in Exp_C regarding the deep learning method and a similar trend was found in the radiomics method (see detailed explanations in “Results” section). It is hard to explain why deep learning performed better in standard convolution kernel images than in bone convolution kernel images to date. We speculate that deep learning networks give their promising performances by learning more discriminative and higher‐level representatives and analysing more comprehensive associations within the tumor. Also, decreasing imaging noise (i.e., using a standard convolution kernel algorithm) may be more important for deep learning networks to learn these higher‐level representatives, instead of increasing spatial resolution (i.e., using bone convolution kernel algorithm). Further validation is necessary to confirm this supposition.

Another interesting finding was that B0 and S60 seemed to have the best performance in bone‐kernel and standard‐kernel models, respectively in the deep learning method. It is well established that the imaging noise decreases logically, as the ASiR strength level increases. This may be the reason why S60 model, the model with minimized imaging noise in our study, achieved the best performance in Exp_C for deep learning. In contrast, B0 model performed better than the other two bone‐kernel models (B30 and B60) in the deep learning method. The bone convolution kernel is famous for being able to better assess tumor heterogeneity (brightness details or textures) than a smoother reconstruction algorithm (i.e., standard convolution kernel) at the sacrifice of increased imaging noise. Of note, the study by Abhishek *et al*. showed that the increase in blurring of images was observed with increasing ASiR strength level, resulting in the decreased number of reproducible radiomic features.^9^ In view of this, retaining a high spatial resolution is more important for facilitating the comprehensive learning of networks than reducing imaging noise‐increasing the ASiR level in the bone convolution kernel.

There were several limitations to our study. First, the sample size was insufficient to perfectly train the model, resulting in the presence of overfitting and instability of the proposed model. Both radiomics and deep learning will benefit from more data in terms of reproducibility and generality, as well as predicting performance. Hence, care should be taken when interpreting these results. Further prospective investigation with an increased sample size is warranted to reach a more persuasive and fairer conclusion. However, the data collected in our study (using the same CT scanner, scanning protocol and reconstructed parameters) is very valuable and rare in clinical practice. Moreover, the included sample size is acceptable for tackling this issue and is greater than in previous studies.^9^, ^11^, ^34^ Second, the distribution of pathological subtypes of lung adenocarcinomas included in this study was unbalanced. Of note is that only one AAH and 24 AIS were included in the current study. However, these lesions are usually diagnosed as benign or indolent and rarely require surgical treatment. To address this dilemma, we categorized AAH, AIS and MIA as one group, which mitigated the influence of data bias. Third, only the GE scanner was used. Hence, variability with other manufacturers' reconstruction algorithms was not included as a comparison in this study. Potential distinctions may be highlighted between two manufacturers, even those with identical scanning parameters. Further investigation is therefore warranted in the future.

## Conclusion

In summary, in our study we demonstrated that the diagnostic performance of deep learning was more susceptible to convolution kernel and iterative reconstruction than radiomics. CT images reconstructed with the standard convolution kernel seem to be more appropriate for imaging analysis. Further investigation with a larger sample size is required.

## Disclosure

The authors declare there is no conflict of interest.

## Supporting information


**Appendix S1.** Supporting Information
