# Supplementary material for: Convolution kernel and iterative reconstruction affect the diagnostic performance of radiomics and deep learning in lung adenocarcinoma pathological subtypes
Source: Thorac Cancer. 2019 Aug 19;10(10):1893–903. doi: 10.1111/1759-7714.13161 (PMC6775016; doi:10.1111/1759-7714.13161)
Supplement: Supplementary file 1 — Appendix S1. Supporting Information [file TCA-10-1893-s001.doc]

**Detailed information about predefined features**

1. First Order Statistics：describe the distribution of voxel intensities within the image region defined by the mask through commonly used and basic metrics；
2. Shape-based: included descriptors of the three-dimensional size and shape of the ROI. These features are independent from the gray level intensity distribution in the ROI and are therefore only calculated on the non-derived image and mask.
3. Gray Level Co-occurence Matrix (GLCM): A GLCM of size *Ng*×*Ng* describes the second-order joint probability function of an image region constrained by the mask and is defined as *P*(*i*, *j* |*δ*,*θ*). The (*i*, *j*)*^th^* element of this matrix represents the number of times the combination of levels *i* and *j* occur in two pixels in the image, that are separated by a distance of *δ* pixels along angle *θ*. The distance *δ* from the center voxel is defined as the distance according to the infinity norm. For *δ*=1, this results in 2 neighbors for each of 13 angles in 3D (26-connectivity) and for *δ*=2 a 98-connectivity (49 unique angles).( note: pyradiomics computes symmetrical GLCM by default)
4. Gray Level Run Length Matrix (GLRLM): A GLRLM quantifies gray level runs, which are defined as the length in number of pixels, of consecutive pixels that have the same gray level value. In a gray level run length matrix *P*(*i*, *j|θ*), the (*i*,*j*)*^th^*  element describes the number of runs with gray level *i* and length *j* occur in the image (ROI) along angle *θ*.
5. Gray Level Size Zone Matrix (GLSZM): A GLSZM quantifies gray level zones in an image. A gray level zone is defined as a the number of connected voxels that share the same gray level intensity. A voxel is considered connected if the distance is 1 according to the infinity norm (26-connected region in a 3D, 8-connected region in 2D). In a gray level size zone matrix P(*i*,*j*)the (*i*,*j*)*^th^* element equals the number of zones with gray level *i* and size *j* appear in image. Contrary to GLCM and GLRLM, the GLSZM is rotation independent, with only one matrix calculated for all directions in the ROI.
6. Neigbouring Gray Tone Difference Matrix (NGTDM): A NGTDM quantifies the difference between a gray value and the average gray value of its neighbours within distance *δ*. The sum of absolute differences for gray level *i* is stored in the matrix.
7. Gray Level Dependence Matrix (GLDM): A GLDM quantifies gray level dependencies in an image. A gray level dependency is defined as a the number of connected voxels within distance *δ* that are dependent on the center voxel.

**The basic architecture and the training process of deep learning network**

The architecture of the proposed DenseSharp Networks is illustrated in the following figure. More details about the hyper-parameters please refer to our previous published paper ^[1]^.


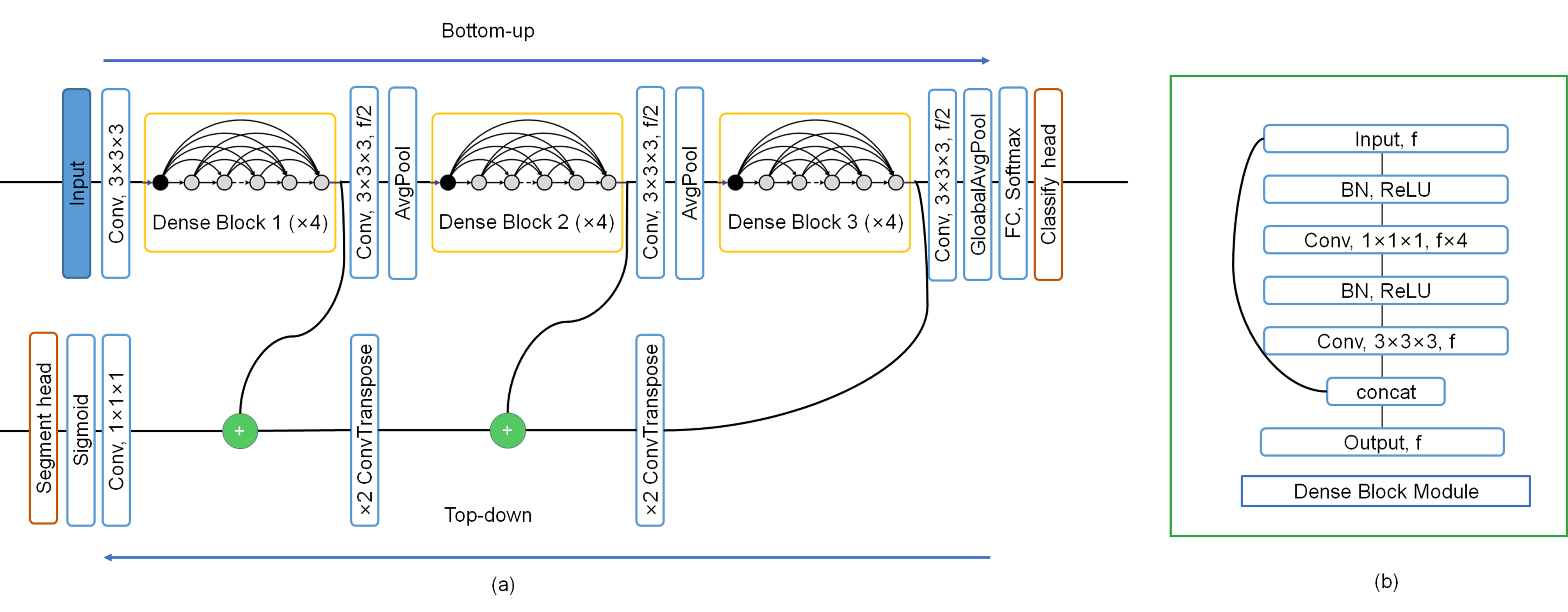


In the process of training deep learning model, the step-size of each epoch was set as 100 (177 samples) in Exp_A and Exp_C-experiment 1. In Exp_B-experiment 2, we set the step-size of each epoch to 200 (1062 samples). The model evaluation metrics included accuracy, recall, precision and fmeasure. During model training, the state and trend of metrics such as learning rate, loss and fmeasure will always be monitored. The initial learning rate was 1e-4. If the loss metric did not decrease after 15 epochs, the learning rate would decrease according to the ratio of 0.334. When the fmeasure metric is no longer upgraded, the model will terminate training early. If the phenomenon of loss divergence was presented, the training will be terminated. These results were obtained in a training process of 40 epochs (see the curve diagram of loss values below). In order to make a fair comparison, the results of 30 training epochs, which showed slight differences in term of loss values corresponding to different models, were selected for further evaluation. Note that investigating the stability other than the best performance of models was our priority in the current study.


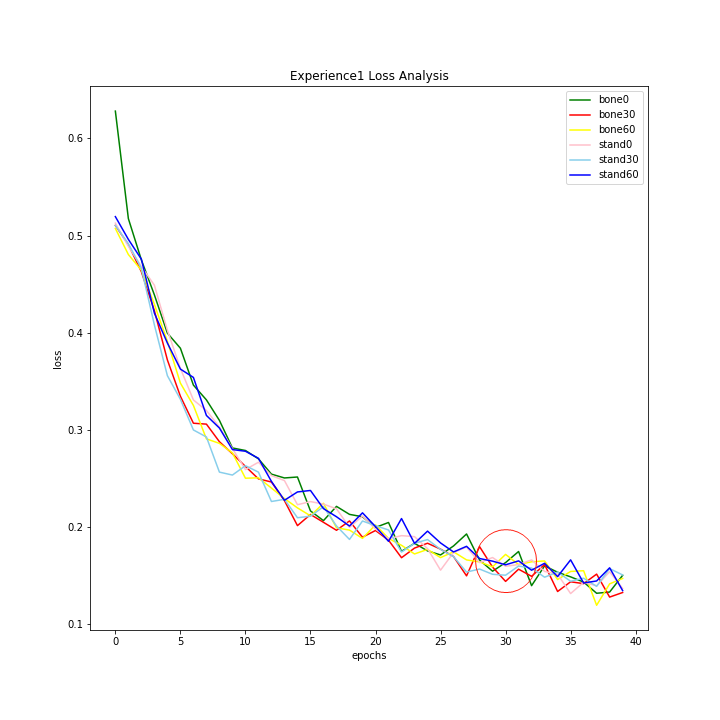


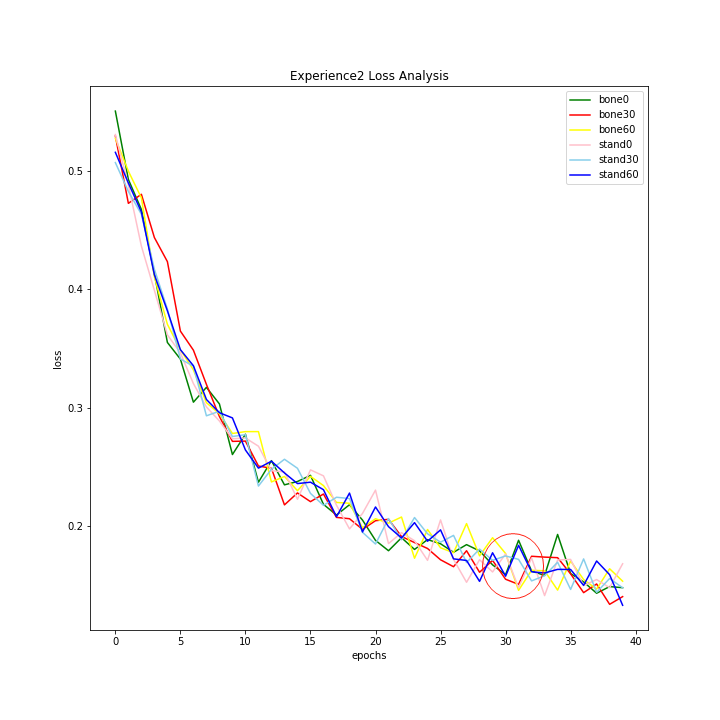


**The illustration of three experiments**

**
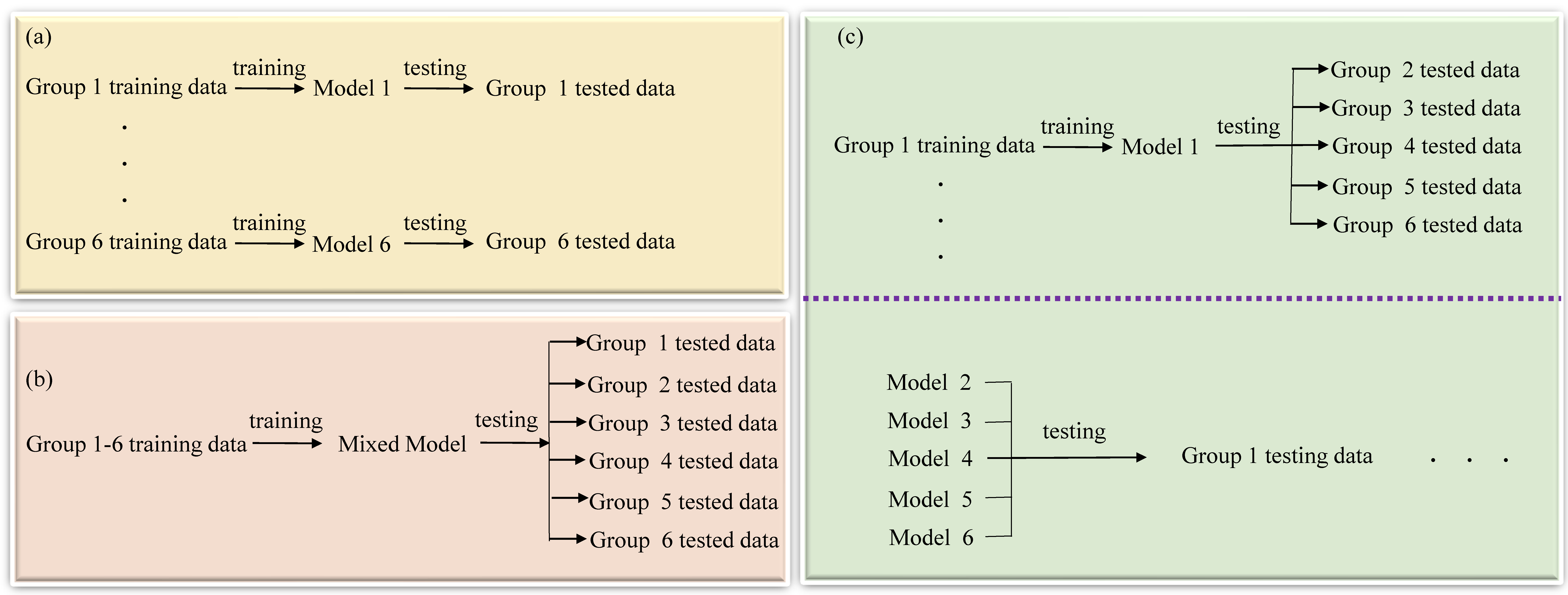
**

**References:**

[1] Zhao W, Yang J, Sun Y, et al. 3D Deep Learning from CT Scans Predicts Tumor Invasiveness of Subcentimeter Pulmonary Adenocarcinomas[J]. Cancer Res. 2018, 78(24): 6881-6889.
